# A Two-Stage Cascade Model of BOLD Responses in Human Visual Cortex

**DOI:** 10.1371/journal.pcbi.1003079

**Published:** 2013-05-30

**Authors:** Kendrick N. Kay, Jonathan Winawer, Ariel Rokem, Aviv Mezer, Brian A. Wandell

**Affiliations:** Department of Psychology, Stanford University, Stanford, California, United States of America; University College London, United Kingdom

## Abstract

Visual neuroscientists have discovered fundamental properties of neural representation through careful analysis of responses to controlled stimuli. Typically, different properties are studied and modeled separately. To integrate our knowledge, it is necessary to build general models that begin with an input image and predict responses to a wide range of stimuli. In this study, we develop a model that accepts an arbitrary band-pass grayscale image as input and predicts blood oxygenation level dependent (BOLD) responses in early visual cortex as output. The model has a cascade architecture, consisting of two stages of linear and nonlinear operations. The first stage involves well-established computations—local oriented filters and divisive normalization—whereas the second stage involves novel computations—compressive spatial summation (a form of normalization) and a variance-like nonlinearity that generates selectivity for second-order contrast. The parameters of the model, which are estimated from BOLD data, vary systematically across visual field maps: compared to primary visual cortex, extrastriate maps generally have larger receptive field size, stronger levels of normalization, and increased selectivity for second-order contrast. Our results provide insight into how stimuli are encoded and transformed in successive stages of visual processing.

## Author Summary

Much has been learned about how stimuli are represented in the visual system from measuring responses to carefully designed stimuli. Typically, different studies focus on different types of stimuli. Making sense of the large array of findings requires integrated models that explain responses to a wide range of stimuli. In this study, we measure functional magnetic resonance imaging (fMRI) responses in early visual cortex to a wide range of band-pass filtered images, and construct a computational model that takes the stimuli as input and predicts the fMRI responses as output. The model has a cascade architecture, consisting of two stages of linear and nonlinear operations. A novel component of the model is a nonlinear operation that generates selectivity for second-order contrast, that is, variations in contrast-energy across the visual field. We find that this nonlinearity is stronger in extrastriate areas V2 and V3 than in primary visual cortex V1. Our results provide insight into how stimuli are encoded and transformed in the visual system.

## Introduction

**Figure 1:**
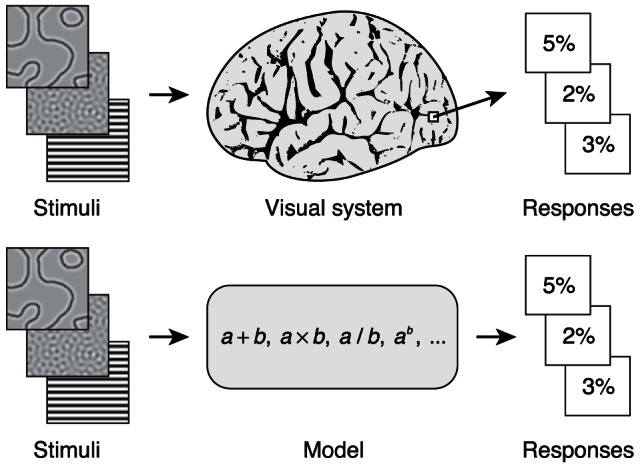
Building general, predictive models of the visual system. We seek to develop computational models that characterize how stimuli are encoded in responses measured in the visual system. These models consist of specific computations and may have parameters that are adjusted to fit the data. Importantly, the models should operate on a wide range of stimuli and predict responses beyond those to which the models are fit.

Studies of visual cortex typically measure responses to a narrow set of stimuli designed to investigate a particular phenomenon. For example, a study might use sinusoidal gratings varying in contrast to study contrast response functions [1], [2], another study might use silhouettes to study shape tuning [3], [4], and yet another study might use arrays of line segments to study texture representation [5], [6]. This approach provides valuable insights, but different effects are studied in isolation and different models (e.g., linear filtering, static nonlinearities, divisive normalization, MAX) are proposed for different effects. To advance our understanding, we seek to develop an integrated model that explains responses to a wide range of stimuli (Figure 1).

In this study, we measure functional magnetic resonance imaging (fMRI) responses in early visual cortex to a wide range of band-pass grayscale images, and we develop a model that starts with images and predicts these responses. The model has a cascade architecture and comprises four main components. The first component is a set of V1-like Gabor filters that are applied to the image. These filters are adapted from our previous work on modeling fMRI responses [7]. The second component is a divisive normalization operation that is applied to filter outputs. Divisive normalization is a well-established computation that accounts for several nonlinear response properties of V1 neurons [8]–[10]. The third component is a compressive static nonlinearity that is applied after summation of contrast-energy across the visual field. We recently found that this nonlinearity is important for accurately predicting responses to stimuli varying in position and size [11]. The fourth component is a variance-like nonlinearity that is used in the summation of contrast-energy. This nonlinearity generates selectivity for second-order contrast and shares some similarities with filter-rectify-filter models that have been proposed for texture perception [12], [13].

We provide software code that implements the complete model along with example datasets at http://kendrickkay.net/socmodel/. This is useful for the goal of reproducible research [14] and provides the opportunity for others to improve upon our work. We welcome efforts to consider potential alternative models—including models developed in psychophysics, computer vision, and the theoretical literature, as well as models that posit specific circuit-level mechanisms—and to determine whether these models better account for the experimental measurements we have made. We hope the open exchange of data and code will spur further modeling efforts.

This paper is structured as follows: We start by motivating each component of our model through targeted examples of stimuli and responses. We then use cross-validation to show that the full model does not overfit the data but in fact improves prediction accuracy. Finally, we examine the parameters of the model and inspect the effect of the parameters on the behavior of the model. This examination reveals that compared to primary visual cortex, extrastriate maps generally have larger receptive field size, stronger levels of normalization, and increased selectivity for second-order contrast.

## Results

We measured blood oxygenation level dependent (BOLD) responses in visual field maps V1, V2, V3, and hV4 while subjects viewed a large number of stimuli. In the main experiment, a total of 156 distinct stimuli were presented in random order 3–6 times each. The BOLD response amplitude of each voxel to each stimulus was estimated from the time-series data using a GLM (see Methods).

### Model motivation

**Figure 2:**
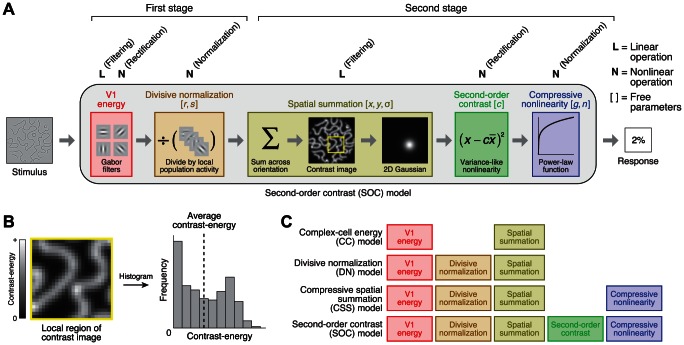
Second-order contrast (SOC) model. (A) Schematic of model. First, the stimulus is filtered with a set of Gabor filters at different positions, orientations, and phases; the outputs of quadrature-phase pairs are squared, summed, and square-rooted (*V1 energy*). Second, filter outputs are divided by local population activity (*Divisive normalization*). Third, filter outputs are summed across orientation, producing a map of local contrast-energy. Contrast-energy is then weighted and summed across space using a 2D Gaussian (*Spatial summation*). The summation is not linear; rather, the summation is performed using a variance-like nonlinearity in which average contrast-energy is subtracted before squaring and summing across space (*Second-order contrast*). Finally, the output of the summation is subjected to a compressive power-law function (*Compressive nonlinearity*), yielding the predicted response. (B) Computation of second-order contrast. Second-order contrast is computed as the variance of the contrast-energy distribution within the 2D Gaussian. In this example, there is high variation in contrast-energy and thus a high amount of second-order contrast. (C) Simplified versions of the model. To motivate the SOC model, we consider several simplified versions of the model. Each version incorporates a model component not present in the previous version.

The model we developed for predicting the BOLD response consists of a sequence of operations (Figure 2A). The BOLD response is predicted by applying V1-like Gabor filters to the luminance image (*V1 energy*), normalizing the filter outputs by local population activity (*Divisive normalization*), summing contrast-energy across a specific region of the visual field (*Spatial summation*) using a variance-like nonlinearity (*Second-order contrast*), and applying a compressive static nonlinearity (*Compressive nonlinearity*). The key novel component of the model is the computation of second-order contrast (Figure 2B), hence the name of the model. The model has eight free parameters (Figure 2A, bracketed variables) and is fit to the response amplitudes of each voxel.

To motivate and explain the second-order contrast (SOC) model, we start with simpler versions of the model and incrementally build up to the full model (Figure 2C). At each step of the process, we assess how well a simple model explains responses to a range of stimuli and improve performance by adding a new component to the model. A caveat to this approach is that increasingly complex models may provide better fits, but these improvements may simply reflect overfitting to the noise in the data. In a later section, we use cross-validation to obtain unbiased estimates of model accuracy and verify that the more complex models are indeed more accurate than the simpler models.

**Figure 3:**
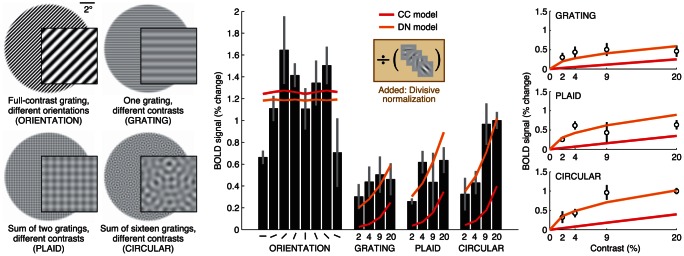
Divisive normalization accounts for contrast saturation. We measured responses to several types of grating stimuli varying in contrast. Responses of an example voxel are shown (subject 2, area V1, voxel 31150). The complex-cell energy (CC) model consists of V1 energy and spatial summation, and predicts that responses rise linearly with contrast. However, the actual responses exhibit saturation at low contrasts. To account for contrast saturation, we incorporated divisive normalization [10] into the model. The divisive normalization (DN) model fits the data accurately.

The simplest model is the complex-cell energy (CC) model, which involves computing V1 energy and summing across the visual field. Previous studies indicate that the CC model is a reasonable starting point: the CC model accounts for substantial variance in BOLD responses in early visual areas to grayscale natural images [7] and a closely related model accurately characterizes BOLD responses to a checkerboard pattern positioned at different visual field locations [15]. For the purposes of this project, the summation weights in the CC model were constrained to be Gaussian across space and equal for different orientations; this is a reasonable approximation for voxel responses [7]. We assessed how well the CC model accounts for responses to a set of stimuli that included oriented gratings and mixtures of oriented gratings presented at different contrast levels (henceforth referred to as *grating stimuli*). Results for an example voxel in V1 are shown (Figure 3).

Responses increase with contrast and with number of orientations, consistent with recent fMRI measurements [16], [17]. This pattern of results is qualitatively reproduced by the CC model (Figure 3, red curve). However, the CC model fails quantitatively: it does not account for the fact that responses tend to saturate at low contrasts. To improve performance, we augmented the CC model with divisive normalization, a computational mechanism that explains a variety of nonlinear behaviors of V1 neurons including contrast saturation [8]–[10]. The divisive normalization (DN) model fits the data accurately (Figure 3, orange curve).

**Figure 4:**
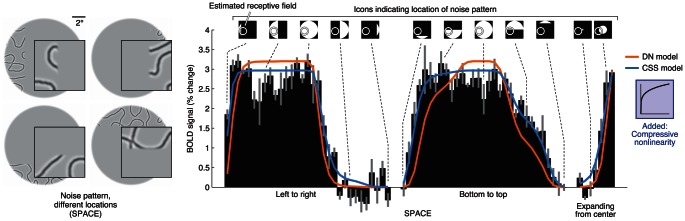
Compressive nonlinearity accounts for spatial tolerance. We measured responses to noise patterns covering different portions of the visual field. Responses of an example voxel are shown (subject 2, area V2, voxel 38512). The DN model underestimates responses to stimuli covering a small portion of the receptive field and overestimates responses to stimuli covering a large portion of the receptive field. To improve performance, we incorporated a compressive static nonlinearity into the model. The compressive nonlinearity is applied after spatial summation and provides increased tolerance for changes in the position and size of a stimulus [11]. The compressive spatial summation (CSS) model fits the data accurately.

To test the DN model on a wider range of stimuli, we measured responses to noise patterns covering different portions of the visual field (henceforth referred to as *spatial stimuli*). Results for an example voxel in V2 are shown (Figure 4). The DN model does a reasonable job capturing the pattern of responses to the stimuli (Figure 4, orange curve). However, the model underestimates responses to stimuli covering a small portion of the receptive field and overestimates responses to stimuli covering a large portion of the receptive field. This can be seen most clearly by inspecting responses to the stimuli labeled ‘Bottom to top’.

We observed this pattern of underestimation and overestimation of spatial responses in a previous study [11] and resolved the issue by applying a compressive static nonlinearity after spatial summation. Intuitively, the compressive nonlinearity boosts responses to stimuli that only partially overlap the receptive field, and can be interpreted as providing tolerance for changes in stimulus position and size [11]. We attempted to improve the performance of the DN model by incorporating, in an analogous fashion, a compressive nonlinearity after spatial summation. We find that the compressive spatial summation (CSS) model better fits the data (Figure 4, blue curve).

**Figure 5:**
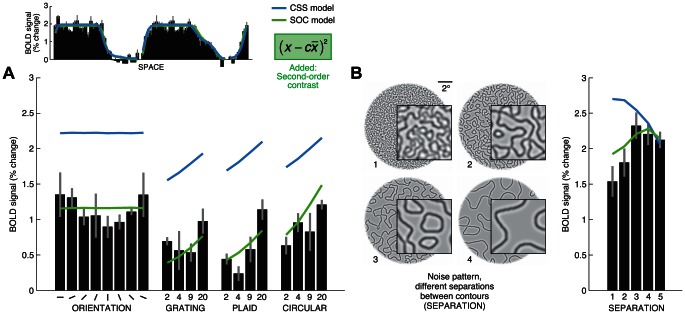
Second-order contrast accounts for weak responses to grating stimuli. (A) Second-order contrast improves model fits. We fit the CSS model to the spatial stimuli (shown in Figure 4) and evaluated how well the model predicts responses to the grating stimuli (shown in Figure 3). Results for an example voxel are shown (subject 2, area V2, voxel 42608). The CSS model substantially overestimates the grating responses. To improve performance, we incorporated computation of second-order contrast into the model. The second-order contrast (SOC) model fits the data accurately. (B) Additional demonstration of second-order effect. We measured responses to noise patterns varying in the amount of separation between the contours composing the patterns. At low separation levels, the stimuli contain little variation in contrast-energy across space and evoke weak responses, as expected (same voxel in panel A).

The CSS model accurately fits responses to the spatial stimuli; and since the CSS model is a more general case of the DN model, the CSS model accurately fits responses to the grating stimuli. However, the CSS model fails to fit responses to the two sets of stimuli simultaneously. For example, if the CSS model is fit to the spatial stimuli, the predicted responses to the grating stimuli substantially overestimate the actual responses (Figure 5A, blue curve). This failure suggests that the CSS model is incomplete and must be modified to account for the full range of responses.

Under the CSS model, the predicted response co-varies with the total amount of contrast-energy within a certain region of the visual field (subject to a compressive nonlinearity). This explains why the model predicts large responses to the grating stimuli, as these stimuli contain contrast-energy throughout the spatial extent of the stimulus. Suppose, however, that BOLD responses are not driven by contrast-energy *per se*, but by variations in contrast-energy. This might explain why the grating stimuli elicit relatively weak BOLD responses.

To improve the performance of the CSS model, we incorporated a variance-like nonlinearity into the spatial summation stage of the model. This nonlinearity suppresses responses to stimuli with spatially homogeneous distributions of contrast-energy and enhances responses to stimuli with spatially heterogeneous contrast-energy distributions. We find that the new model, which is the full second-order contrast (SOC) model, simultaneously fits both the spatial stimuli and the grating stimuli (Figure 5A, green curve).

The noise patterns used for the spatial stimuli consist of contours that are spatially separated from one another; this spatial separation gives rise to variation in contrast-energy and generates large responses from the SOC model. We hypothesized that reducing the spatial separation of the contours would reduce variation in contrast-energy and lead to reduced BOLD responses. To test this hypothesis we measured responses to noise patterns with different levels of contour separation (Figure 5B). As expected, we find that the response is lowest at the smallest separation and increases at larger separations. This pattern of results is accurately predicted by the SOC model (Figure 5B, green curve) but not the CSS model (Figure 5B, blue curve).

### Model evaluation

To systematically evaluate the merit of the SOC model, we fit that model and each of the simpler models (CC, DN, CSS) independently to the data using five-fold cross-validation. Cross-validation produces a *prediction* of each data point based on a model that is not fit to that data point. Models are evaluated by how well model predictions match the data.

Because the SOC model subsumes the simpler models, it is guaranteed to produce the best fits for a given set of data. However, there is no guarantee that the SOC model will cross-validate well, i.e. generalize to unseen data. The SOC model will cross-validate well only if the effects described by the model are sufficiently large and there are sufficient data to estimate model parameters accurately. Cross-validation controls for model complexity since overly complex models will tend to fit noise in the data and, as a result, generalize poorly. Alternative methods for model selection include AIC and BIC, and these methods produce similar results (see Supporting Figure S1).

**Figure 6:**
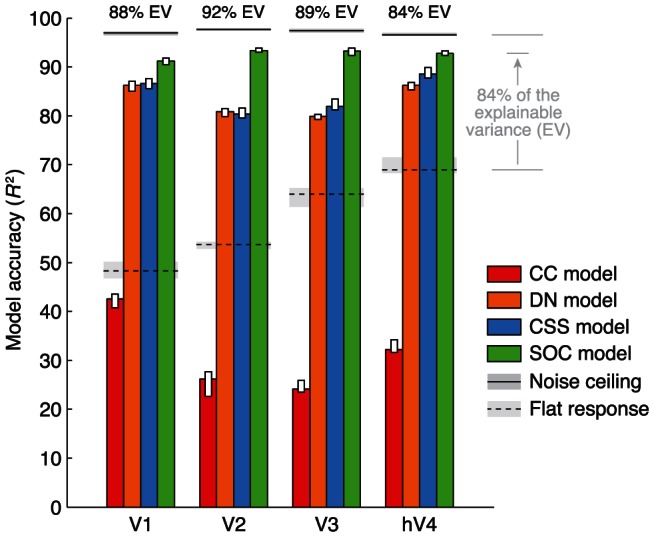
SOC model has high cross-validation accuracy. Five-fold cross-validation was used to quantify the accuracy of the CC, DN, CSS, and SOC models. Vertical bars indicate the median accuracy across voxels in a given visual field map. Solid horizontal lines indicate the maximum possible performance given the noise in the data, and dotted horizontal lines indicate the performance of a control model that simply predicts the same response for every stimulus. The numbers at the top indicate the median performance of the SOC model, expressed in terms of explainable variance (see Methods). Within each visual field map, all pairwise differences between models are statistically significant (*p*<0.05, two-tailed sign test) with the exception of DN vs. CSS in V2.

In all visual field maps, we find that the SOC model has the highest cross-validation accuracy (Figure 6). The accuracy of the SOC model is slightly lower than the noise ceiling, i.e., the maximum performance that can be expected given the noise in the data. Using the metric of explainable variance which takes into account the noise ceiling (see Methods), we find that on average, the SOC model accounts for 88%, 92%, 89%, and 84% of the explainable variance in V1, V2, V3, and hV4, respectively (median across voxels in each map). These values indicate the high predictive power of the SOC model.

Metrics like variance explained are convenient for summarizing model accuracy, but it is important to examine the specific aspects of the data that drive these metrics. To visualize results from a large number of voxels on a single plot, we adopt the strategy of averaging data across voxels and averaging the predictions of each model across voxels. Note that this averaging is only for sake of visualization; cross-validation accuracy is computed on a voxel-by-voxel basis and does not involve averaging data.

**Figure 7:**
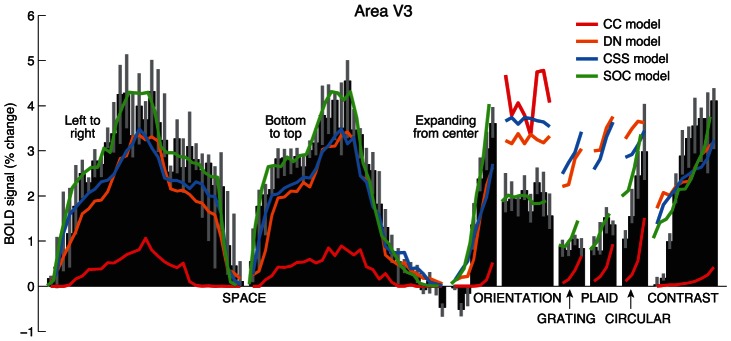
Data and cross-validated model predictions. Here we visualize the cross-validation results by averaging across voxels in a visual field map. Black bars indicate the median response across voxels, and colored curves indicate the median model prediction across voxels. The CC model captures qualitative features of the data but fails quantitatively. The DN and CSS models fare better than the CC model but systematically underestimate and overestimate certain responses. The SOC model does well quantitatively predicting the full range of responses.

Examining the data and model predictions for a representative visual field map, we see that the SOC model clearly outperforms the other models (Figure 7). In interpreting this plot, keep in mind that the predictions of a model may depend on the specific stimuli to which the model is fit. For example, when fit to a wide range of stimuli, the DN model fails to predict responses to the grating stimuli (Figure 7, orange curve), despite the fact that the DN model succeeds when the model is fit only to the grating stimuli (see Figure 3). As another example, the CC model performs quite poorly for the stimuli tested in this study (Figure 7, red curve), which may seem surprising given previous reports that the CC model (or variants thereof) can characterize responses to grayscale natural images [7] and retinotopic mapping stimuli [15]. However, the results are not inconsistent. The key realization is that the CC model may perform well if fit and tested on stimuli that probe a limited range of stimulus dimensions (e.g. a limited range of contrasts). With a wide range of stimuli, failures of the CC model become evident, and more complex models are necessary to explain the data.

We developed the SOC model using carefully controlled stimuli and have demonstrated that the model accurately characterizes responses to these stimuli. A major advantage of controlled stimuli is ease of interpretation: with controlled stimuli, it is relatively easy to identify the stimulus properties that drive effects in the data [18]. However, a stimulus set composed of controlled stimuli is inherently biased towards certain stimulus types at the exclusion of others, leaving open the question of how well the model characterizes responses to stimuli in general.

To estimate general accuracy, in a separate experiment we measured responses to 35 objects and quantified how well the SOC model—with parameters derived from the controlled stimuli—predicts the responses. On average, the SOC model accounts for 65%, 72%, 69%, and 59% of the explainable variance in V1, V2, V3, and hV4, respectively (median across voxels in each map). These values are lower than the corresponding values obtained for the controlled stimuli, underscoring the fact that summary metrics of model performance are highly dependent on the type of stimuli used. Nevertheless, the values are encouragingly high and confirm that the SOC model has predictive power for ecologically relevant stimuli [19]. One interpretation of the reduced performance on object stimuli is that such stimuli contain higher-order features that are not accurately represented by the SOC model; investigating these features can be the focus of future studies.

In the divisive normalization stage of the SOC model, the population activity used to normalize filter outputs consists of the sum of the outputs of filters at the same position but different orientations (see Methods). The reason we assumed the population has the same spatial extent as the filter outputs is simplicity: by making that assumption, the space of model parameters is vastly reduced and the interpretation of the divisive normalization stage is simplified. However, divisive normalization models of V1 neurons often consist of a central excitatory region that is normalized by a larger surround region [20], [21], and such models are used to account for surround suppression, a phenomenon that is closely related to second-order contrast (see Discussion). Thus, one might speculate that if the spatial extent of the population were enlarged, the resulting model might be sufficient to account for our data.

**Figure 8:**
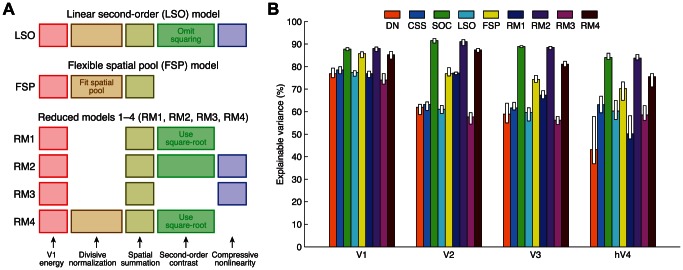
Additional control models. (A) Schematic of models. Six variants of the SOC model were tested. Text annotations indicate modifications to model components (see Methods for details). (B) Cross-validation accuracy. Format same as in Figure 6 except that accuracy is now expressed in terms of explainable variance (the CC model is omitted as it falls outside the visible range). No model outperforms the SOC model. The RM2 model—which is a variant of the SOC model that omits the *Divisive normalization* component—performs about as well as the SOC model. This can be explained by the fact that there is some degree of overlap in functionality between the *Divisive normalization* and *Compressive nonlinearity* components of the SOC model.

To address this issue, we tested a version of the DN model in which the spatial scale over which normalization occurs is flexible and fit to the data. The hypothesis is that this model might account for the data as well as (or better than) the more complex SOC model. We find that the DN model with flexible normalization (Figure 8B, yellow bar) outperforms the original DN model (Figure 8B, orange bar) but does not achieve the same accuracy as the SOC model (Figure 8B, green bar). This indicates that simply enlarging the normalization pool is not sufficient and that the additional computations in the SOC model are necessary to account for the data. We also tested several other control models, including a model that demonstrates that the squaring operation in the computation of second-order contrast is critical (Figure 8B, cyan bar).

One of the control models (RM2) omits the *Divisive normalization* component of the SOC model, and performs about as well as the full SOC model. This can be attributed to the fact that the effect of *Divisive normalization* on the overall response of the model can be approximated, through suitable choice of parameters, by the other components of the model, most notably the *Compressive nonlinearity* component. For a simple example of this phenomenon, suppose we have a cascade of two power-law nonlinearities, each with exponent 0.5. If the first nonlinearity is omitted, the overall input-output relationship can still be preserved if the exponent of the second nonlinearity is set to 0.25. While a compressive nonlinearity is not an exact substitute for divisive normalization, it approximates many of the same effects within our measurements. We have chosen to include the *Divisive normalization* component in the SOC model for two reasons. One is to maintain historical continuity, as previous studies have incorporated divisive normalization immediately following a linear filtering stage [e.g. 9]. The second reason is that even though normalization (immediately after the linear filtering stage) is not essential for the current set of data, it is likely that normalization will prove essential at finer scales of measurement (sub-millimeter voxels). For example, a major effect explained by normalization is cross-orientation suppression at the level of single neurons in V1 [10]; this effect is largely obscured at the current scale of measurement (2.5-mm voxels). This observation highlights the fact that the model inferences we make are limited by the resolution of our BOLD measurements and that there is value in developing models at finer scales of measurement.

### Model parameters

We now turn to examining the parameters of the SOC model. There are three parameters of interest, σ, *n*, and *c*. The σ parameter controls the size of the 2D Gaussian over which contrast-energy is summed, the *n* parameter controls the strength of the compressive nonlinearity, and the *c* parameter controls the strength of the variance-like nonlinearity that generates selectivity for second-order contrast (see Methods for details). To summarize the *n* and *c* parameters, we calculate the median parameter value across voxels in each map. To summarize the σ parameter, we fit a line relating receptive field eccentricity and σ and extract the σ value at 2° eccentricity.

**Figure 9:**
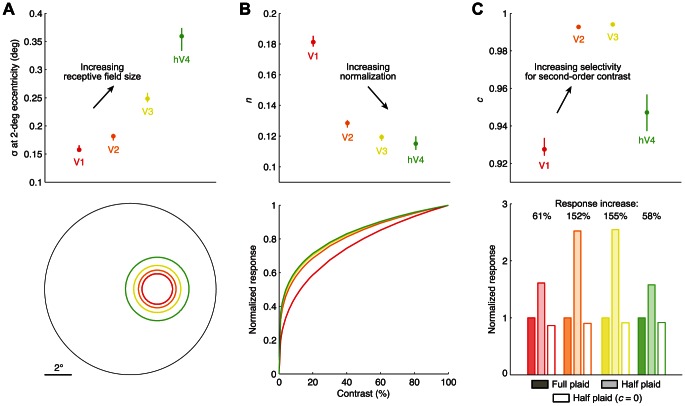
Parameters of the SOC model vary systematically across visual field maps. (A) Size parameter (σ). The top panel shows the estimated σ value at 2° eccentricity for each visual field map. To quantify receptive field size, we compute model responses to small white spots (0.25°×0.25°) and fit 2D Gaussians to the results. The bottom panel shows contours at ±2 s.d. of the fitted Gaussians. (B) Exponent parameter (*n*). The top panel shows the median *n* value for each visual field map. To demonstrate the effect of *n*, we compute model responses to full-field noise patterns varying in contrast (same patterns used for the spatial stimuli). The bottom panel shows the resulting contrast response functions, normalized such that the maximum response is 1. (C) Second-order parameter (*c*). The top panel shows the median *c* value for each visual field map. To interpret the effect of *c*, we compute model responses to a 20%-contrast plaid pattern covering the entire receptive field and the same plaid pattern covering half of the receptive field. The bottom panel shows responses to the full and half plaids, normalized such that the response to the full plaid is 1. For reference we also show results obtained when *c* is set to 0.

For each parameter of interest, we plot the summary value observed in each visual field map (Figure 9, top). Because raw parameter values are difficult to interpret, we also perform simulations that clarify the effect of the parameter values on the overall stimulus-response relationship (Figure 9, bottom). In these simulations, we calculate the response of the SOC model using the typical parameter values found in each visual field map (thus, four instances of the SOC model were simulated). These simulations directly reflect the behavior of the SOC model as fitted to each map and do not incorporate any assumptions beyond what is determined from the data and the model.

Inspecting the variation in parameter values, we find that the σ parameter increases from V1 to V2 to V3 to hV4, reflecting an increase in receptive field size (Figure 9A). We find that the *n* parameter decreases from V1 to V2 to V3 to hV4, reflecting an increase in normalization (Figure 9B). Finally, we find that the *c* parameter is higher in V2 and V3 than it is in V1 and hV4, reflecting increased selectivity for second-order contrast (Figure 9C). All pairwise differences between visual field maps are statistically significant (*p*<0.05, two-tailed randomization test) with the exception of *n* in V3 vs. hV4 and *c* in V2 vs. V3.

## Discussion

We describe a computational model, termed the second-order contrast (SOC) model, that predicts BOLD responses in early visual cortex to grayscale band-pass filtered images. The model builds on earlier modeling work [7], [10], [15] and introduces a variance-like nonlinearity that generates selectivity for second-order contrast. The parameters of the model vary systematically across visual field maps, reflecting differences in receptive field size, differences in the strength of normalization, and differences in selectivity for second-order contrast.

### Building functional models of visual responses

We have developed a model that predicts BOLD responses to a wide range of stimuli. Stimulus-driven BOLD responses arise principally from metabolic demands of peri-synaptic neural activity [22], [23]. Hence, BOLD is one of the many ways that neural activity can be measured, and our model of BOLD responses is a model of neural population responses. However, the spatial resolution of our BOLD measurements (2.5-mm voxels) is lower than the resolution required to analyze and dissect neural circuits, and this may lead some to conclude that our model of BOLD responses does not actually provide much insight into neural computation. We believe this view to be in error.

To explain our position, it is useful to highlight the distinction between *functional models* and *circuit models*. Functional models are stimulus-referred (i.e. start with the stimulus) and specify what aspects of the stimulus drive responses in a given area. Building functional models has a long history in electrophysiology [for review], , where researchers explain the spiking activity of neurons in terms of relatively simple computations applied to the stimulus. Circuit models go further than functional models by identifying the specific neural circuitry that gives rise to the observed responses. Hence, functional models may be simpler than circuit models and multiple competing circuit models may be consistent with a given functional model. There is value in functional characterizations of neural responses, especially if one seeks to link neural circuits to perceptual judgments and behavior [26].

To illustrate the distinction between functional models and circuit models, consider a model that explains the spiking activity of a V1 simple cell by the application of an oriented linear filter to the stimulus, followed by a rectification nonlinearity. This model, known as an LN or linear-nonlinear model [24], is a functional but not a circuit model—it describes how stimuli relate to responses, but does not characterize the many stages of processing performed by the visual system before V1 (e.g. retina, LGN) nor the specific neural circuit by which orientation tuning arises [e.g. feedforward computation on LGN afferents or intracortical processing within V1—see 27]. Nevertheless, the model is useful for understanding how stimuli are represented in the visual system.

The SOC model developed in this paper is a functional model—it characterizes the relationship between visual stimuli and measured BOLD responses. Like functional models of neuronal responses, the SOC model does not propose specific neural circuits. Rather, the SOC model provides insight at the functional level, that is, in identifying the aspects of the stimulus that drive responses in different visual field maps. For example, the model indicates that second-order contrast is an important factor that drives population responses in V2 and V3, and we can reasonably infer that this same stimulus property drives responses of individual neurons in these maps. To test and expand upon this hypothesis, one could adapt the stimuli and model used in this study to single-unit electrophysiology and assess how well neuronal responses are accounted for. In doing so, we may find it necessary to extend the model to account for response properties that are evident at the level of individual neurons but which are not readily observed at the population level.

### Nonlinearities in neurovascular coupling

Neural activity is coupled to the BOLD response through a complex set of neurovascular mechanisms [23], [28]. Thus, physiological responses measured using BOLD fMRI reflect both neural activity and these coupling mechanisms. Since the coupling mechanisms are not explicitly modeled in the present work, an implicit assumption in the interpretation of our results is that the BOLD response provides a linear (or approximately linear) measure of some aggregated neural activity. Under this assumption, we attribute the various nonlinear operations in the SOC model to nonlinearities arising in neural processing. However, there may be nonlinearities in neurovascular coupling, and this possibility limits the inferences we can make from our BOLD measurements. For example, if there is a nonlinearity in the relationship between the total amount of neural activity in a voxel and the strength of the BOLD response measured from that voxel, then the level of compression estimated by the *Compressive nonlinearity* component of the SOC model may differ from the level of compression associated with the underlying neural activity. Going forward, we believe that developing a better understanding of the different types of neural activity (e.g. synaptic activity, spiking activity) and the mechanisms that couple these various types of neural activity to the BOLD response is of high importance.

### Cascade architecture of the SOC model

The SOC model has a cascade architecture, consisting of a series of computations that are applied to the stimulus. The success of the SOC model is consistent with the long-standing hypothesis that the visual system can be characterized as a cascade of operations [29]–[34]. However, cascade models come in a variety of different forms and vary in essential characteristics such as the number of stages in the model and the computations that are applied at each stage. Our work contributes to the field by proposing a specific model and showing that this model quantitatively accounts for a sizable range of experimental measurements in the living human brain.

The SOC model is most similar to the cascade model that is being developed by Heeger, Landy, and colleagues [34], [35]. These authors propose that the stimulus is transformed through two or more stages of canonical operations, each stage consisting of filtering, which is a linear operation (L); rectification, which is a nonlinear operation (N); and normalization, which is a nonlinear operation (N). Mapping these operations onto the SOC model, we see that the SOC model is a two-stage cascade model with an overall form of LNNLNN (Figure 2A).

There are differences between the SOC model and the Heeger-Landy model. First, the SOC model is fully computable, starting with images and predicting physiological responses. Second, the filtering operation in the second stage of the SOC model is generic: variance in contrast-energy drives responses irrespective of how contrast-energy is arranged in the stimulus. In contrast, the Heeger-Landy model uses oriented second-order filters. Third, the normalization operation in the second stage of the SOC model is implemented as a compressive nonlinearity. This is reasonable because under certain conditions, the effects of divisive normalization can be approximated with a compressive nonlinearity [11].

It is common in cascade models to designate different stages as corresponding to different visual areas. Thus, it is tempting to view the first stage of operations in the SOC model (the first LNN) as corresponding to primary visual cortex (V1) and the second stage of operations (the second LNN) as corresponding to extrastriate areas. However, this interpretation is complicated by the fact that the full two-stage SOC model predicts V1 responses more accurately than the one-stage DN model (see Figure 6). To reconcile this finding, we hypothesize that the computation of first-order contrast (the first LNN) occurs in V1 (or is inherited from earlier processing), the computation of second-order contrast (the second LNN) occurs downstream from V1, and feedback introduces second-order effects into V1 responses. Some support for this circuit-level hypothesis comes from studies reporting that surround suppression—which, as we later explain, is intimately related to second-order contrast—is mediated by feedback from extrastriate areas to V1 [36], [37].

### Second-order contrast in the visual system

A key component of the SOC model is a nonlinearity that computes variance in contrast-energy within a specific region of the visual field. This nonlinearity enhances responses to stimuli that have heterogeneous distribution of contrast-energy and suppresses responses to stimuli that have homogeneous distribution of contrast-energy. We find that the nonlinearity is substantially stronger in extrastriate areas V2 and V3 compared to V1, suggesting that selectivity for second-order contrast is mainly a feature of extrastriate cortex. We do find, however, that the strength of the nonlinearity in hV4 is comparable to that in V1, indicating that in hV4 first-order contrast is relatively effective at driving responses.

The concept of second-order contrast—or, more generally, second-order stimuli—has a long history in visual psychophysics [for review], [ see 12,13] and other sensory modalities [38]. Second-order stimuli involve modulation of a stimulus property (e.g. contrast) across space or time in such a way that the modulation cannot be detected by a first-order filter. For example, consider a sinusoidal grating whose amplitude is modulated by a sinusoidal grating of lower spatial frequency. Such a stimulus varies in contrast across space, but this variation cannot be detected by a first-order luminance filter since average luminance remains constant throughout the extent of the stimulus. To explain the perception of second-order stimuli, researchers have proposed filter-rectify-filter (FRF) models in which first-order filters are applied to the stimulus, the outputs of these filters are rectified, and second-order filters are applied to the rectified outputs.

Extending results from animal models [e.g. 39], [40], [41], several fMRI studies have found evidence of second-order processing in human visual cortex [35], [42]. These studies used adaptation techniques to infer selectivity for second-order modulation of contrast [42] and orientation [35], [42], and proposed a variant of the FRF model to account for their results [35]. Our results are consistent with these adaptation studies in finding that second-order effects exist in many visual field maps including V1. We extend these studies by executing a different experimental and modeling approach: We demonstrate second-order effects directly in visually evoked responses. Moreover, we develop a model that operates on images and quantitatively predicts responses at the level of single voxels.

Our finding that selectivity for second-order contrast is particularly strong in extrastriate areas is consistent with the fact that sparsely distributed contours strongly activate such areas [43]. This is because sparsely distributed contours give rise to large amounts of contrast variation. Our results are also consistent with the results of a study that developed and compared models of neural responses in V1 and V2 [44]. In that study, neural responses were characterized using a model in which V1-like filters are applied to the stimulus, the outputs of the filters are rectified, and then a flexible set of weights on the rectified filter outputs is used to predict responses. Importantly, fitted weights tended to be more negative in V2 than in V1. This suppression may serve to reduce responses to stimuli that are spatially homogeneous in contrast-energy, similar to the variance-like nonlinearity we propose in the SOC model. A quantitative comparison of these models is an important future direction.

### Relationship between second-order contrast and surround suppression

**Figure 10:**
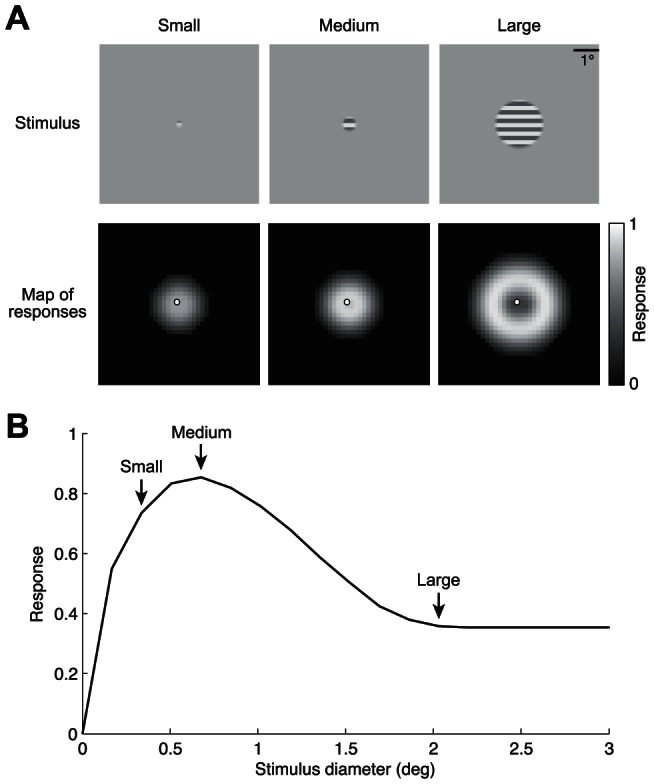
SOC model exhibits surround suppression. (A) Simulation results. Stimuli consisted of a horizontal grating presented within circles of different sizes. Using the typical parameter values found in V2 (see Figure 9), we simulated the response of an array of model units tiling the visual field. Responses are strongest for units positioned at the edge of the grating since responses are driven primarily by variation in contrast-energy. (B) Responses of one unit (marked by a white dot in panel A). With increasing stimulus size, the response rises and then falls, consistent with surround-suppression effects found in electrophysiology [e.g. 20], [48].

Second-order contrast is a key feature of the SOC model, and it is useful to clarify the connection between second-order contrast and phenomena that have been extensively studied in the visual system. One such phenomenon is surround suppression, which has been studied both psychophysically and physiologically and is thought to underlie perceptual processes such as scene segmentation [45], perceptual constancies [46], and enhancement of salience differences [47]. A basic form of surround suppression is size tuning, whereby the response of a neuron is highest for a grating of a certain size and is suppressed if the grating is enlarged [20], [48]. The SOC model was not specifically designed to account for size tuning, but a simulation demonstrates that the SOC model does in fact exhibit size tuning (Figure 10). Intuitively, response suppression for large gratings stems from the absence of variation in contrast-energy; conversely, response enhancement for small gratings stems from the presence of variation in contrast-energy. This simulation demonstrates the close relationship between second-order contrast and surround suppression.

The SOC model's explanation of surround suppression differs from that provided by traditional models of surround suppression. In such models, a central excitatory region is divisively normalized by a larger surround region, and response suppression for large gratings stems from increased stimulation of the surround [20], [21]. The fact that surround suppression might have different computational explanations—either divisive normalization over a large spatial extent or second-order mechanisms—has been previously recognized [35]. We find that divisive normalization by itself does not fully account for our data, even if the spatial extent of normalization is enlarged (see Figure 8B, yellow bar). Thus, our results suggest that second-order mechanisms play an essential role in producing surround suppression effects. The ability to tease apart computational explanations such as these is made possible by our approach of measuring responses to a wide range of stimuli and testing general models that operate on arbitrary stimuli.

### Prevalence of second-order contrast in natural images

Second-order contrast also has an interesting connection to the statistics of natural images. The distribution of local contrast in a natural image tends to be sparse, with local contrast often near zero [50]–[53]. We reasoned that because of this sparseness, the amount of second-order contrast in natural images should be relatively high. To verify this hypothesis, we constructed a collection of natural image patches and quantified the amount of second-order contrast in each image by computing the response of the SOC model to the image. For comparison we also computed responses of the SOC model after scrambling the phase spectrum of each patch.

**Figure 11:**
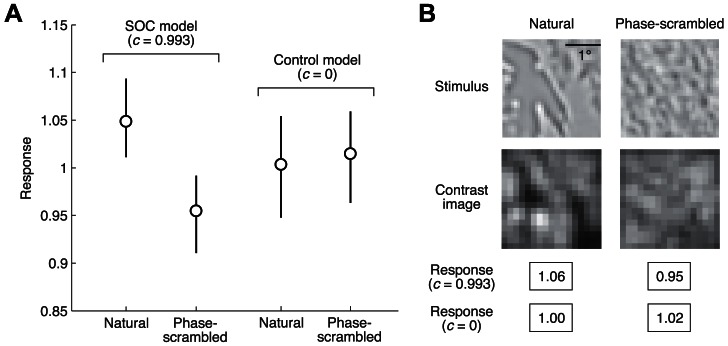
Natural images have relatively large amounts of second-order contrast. (A) Simulation results. We prepared a collection of band-pass filtered natural image patches and phase-scrambled versions of these patches. We then quantified the amount of second-order contrast in each patch by computing the response of the SOC model to the patch (model parameters were set to the typical values found in V2). The median and interquartile range of responses are shown. For comparison we show results obtained when the second-order parameter *c* is set to 0. The SOC model but not the control model exhibits larger responses to the natural image patches. (B) Example patches. The natural image patch exhibits spatial variation in contrast, whereas its phase-scrambled counterpart is relatively homogeneous in contrast across space. Natural images were obtained from the McGill Colour Image Database [73].

The responses of the SOC model are, on average, higher for the natural image patches (Figure 11A). Reduced responses to the phase-scrambled patches can be attributed to the fact that phase-scrambling takes localized structures (which induce high variation in contrast-energy) and disperses them throughout the image (Figure 11B). The fact that natural stimuli have relatively high amounts of second-order contrast is consistent with previous analyses of natural image statistics [54]. We suggest that selectivity for second-order contrast can be interpreted as an efficient coding strategy in which the visual system is tuned to the statistical features of natural scenes [49]. Stated simply, the idea is that the visual system is tuned in such a way that commonly experienced stimuli (e.g. stimuli with second-order contrast) evoke stronger responses than less commonly experienced stimuli (e.g. stimuli without second-order contrast).

Our simulations show that scrambling the phase spectra of natural image patches reduces variation in contrast-energy and leads to reduced responses from the SOC model. In general, reduction in contrast-energy variation may explain why phase scrambling tends to reduce activation levels in the visual system. For example, phase-scrambling line and edge stimuli reduces variation in contrast-energy and, as expected, reduces BOLD responses in early visual areas [55]. Of course, the phase spectrum consists of other stimulus characteristics besides variation in contrast-energy, and the visual system might also be sensitive to these characteristics. One example is alignment of phases across spatial frequencies, which occurs at edges in natural images [56].

### Future improvements to the SOC model

The SOC model has high accuracy but is not perfect, especially when tested on naturalistic object stimuli (see Results). To improve performance, future work could continue the approach taken in the present study of designing controlled stimuli, assessing model predictions, and introducing new model components as necessary. It may be productive to consider how well the SOC model predicts responses to simple icons and shapes as such stimuli have been previously used to study the tuning properties of extrastriate areas [57]–[59].

Future work could also be directed towards expanding the range of stimuli for which the SOC model operates. For tractability we restricted the stimuli in this study to a band-pass range of spatial frequencies. A natural step would be to extend the SOC model to operate on stimuli with arbitrary spatial frequency content. This could be done, for example, by replicating the model architecture at multiple spatial scales and allowing the predicted response to be a weighted sum across scales. Ultimately, additional stimulus properties such as color, motion, and depth will need to be considered.

## Methods

### Subjects

Three experienced fMRI subjects (three males; age range 29–39; mean age 33) participated in this study. All subjects had normal or corrected-to-normal visual acuity. Informed written consent was obtained from all subjects, and the experimental protocol was approved by the Stanford University Institutional Review Board. One subject (JW) was an author. Subjects participated in 1–2 scan sessions for the main experiment, and one subject participated in an additional scan session for the object experiment. Subjects also participated in 1–4 separate scan sessions to identify visual field maps [details in 60].

### Visual stimuli

#### Display and task

Stimuli were presented using a Samsung SyncMaster 305T LCD monitor positioned at the head of the scanner bed. Subjects viewed the monitor via a mirror mounted on the RF coil. The monitor operated at a resolution of 1280×800 at 60 Hz, and the luminance response of the monitor was linearized using a lookup table based on spectrophotometer measurements (maximum luminance 117 cd/m^2^). Stimuli subtended 12.5–12.8° of visual angle (viewing distance 179–183 cm). A MacBook Pro computer controlled display calibration and stimulus presentation using code based on Psychophysics Toolbox [61], [62]. Behavioral responses were recorded using a button box.

For subject 1, a small dot (0.1°×0.1°) at the center of the stimulus served as the fixation point. The color of the dot changed randomly between red, green, and blue every 5–9 s. The subject was instructed to fixate the dot and to press a button whenever the dot changed color. For subjects 2–3, a more demanding attentional task was used [35]. A small digit (0.25°×0.25°) at the center of the stimulus served as the fixation point. The identity of the digit (0–9) changed every 0.67 s: each digit was presented for 0.5 s and was followed by a delay of 0.17 s. To minimize visual adaptation, the digit color alternated between black and white on successive presentations. Subjects were instructed to fixate the digit and to press a button whenever the same digit repeated. Digit repetitions occurred with a probability of 1/6, with a maximum of two successive identical digits allowed.

#### General stimulus characteristics

Stimuli were constructed at a resolution of 256 pixels×256 pixels and were upsampled to 800 pixels×800 pixels for display purposes. All stimuli were presented within a circular aperture filling the height of the display; the rest of the display was filled with neutral gray. The outer 0.5° of the circular aperture was smoothly blended into the background using a half-cosine function.

Stimuli consisted of grayscale images restricted to a band-pass range of spatial frequencies centered at 3 cycles per degree. To enforce this restriction, a custom band-pass filter was used in the generation of some of the stimuli. The filter was a zero-mean isotropic 2D Difference-of-Gaussians filter whose amplitude spectrum peaks at 3 cycles per degree and drops to half-maximum at 1.4 and 4.7 cycles per degree. Restricting the spatial frequency content of the stimuli avoids the complications of building multi-scale models and helps constrain the scope of the modeling endeavor. Even with the spatial frequency restriction, it is possible to construct a rich diversity of stimuli including objects and other naturalistic stimuli.

#### Main experiment (subjects 1–3)

This experiment consisted of 156 stimuli. Data corresponding to 103 of the stimuli are reported in this paper; data corresponding to the remaining 53 stimuli are not used and therefore not described in further detail. Each stimulus consisted of nine distinct images that were presented in quick succession. The purpose of this design was to take advantage of the slow dynamics of the BOLD response and average over stimulus dimensions of no interest (e.g. using sinusoidal gratings differing in phase to average over phase).

*SPACE (69 stimuli).* These stimuli consisted of noise patterns covering different portions of the visual field. Noise patterns were created by low-pass filtering white noise at a cutoff frequency of 0.5 cycles per degree, thresholding the result, performing edge detection using derivative filters, inverting image polarity such that edges are black, and applying the custom band-pass filter (described previously). We generated nine distinct noise patterns and scaled the contrast of the patterns to fill the full luminance range. We then varied the location of the noise patterns by masking the patterns with spatial apertures. The design of the apertures was identical to that used in a previous study [11]. A total of 69 apertures were used: 31 vertical apertures proceeding left to right, 31 horizontal apertures proceeding bottom to top, and 7 circular apertures expanding in size from the center. To maintain the band-pass characteristic of the stimuli, aperture edges were smoothly transitioned into the background using half-cosine functions 1/6° in width.

*ORIENTATION (8 stimuli).* These stimuli consisted of full-contrast sinusoidal gratings at eight different orientations. The spatial frequency of the gratings was fixed at 3 cycles per degree. Each stimulus consisted of gratings with the same orientation but nine different phases (equally spaced from 0 to 2π).

*GRATING (4 stimuli).* These stimuli consisted of horizontal sinusoidal gratings at 2%, 4%, 9%, and 20% Michelson contrast. The spatial frequency of the gratings was fixed at 3 cycles per degree. Each stimulus consisted of gratings with the same contrast but nine different phases (equally spaced from 0 to 2π).

*PLAID (4 stimuli).* These stimuli consisted of plaids at 2%, 4%, 9%, and 20% contrast (defined below). Each condition comprised nine plaids, and each plaid was constructed as the sum of a horizontal and a vertical sinusoidal grating (spatial frequency 3 cycles per degree, random phase). The plaids were scaled in contrast to match the root-mean-square (RMS) contrast of the GRATING stimuli. For example, the plaids in the 9% condition were scaled such that the average RMS contrast of the plaids is identical to the average RMS contrast of the gratings in the 9% GRATING stimulus.

*CIRCULAR (4 stimuli).* These stimuli were identical to the PLAID stimuli except that sixteen different orientations were used instead of two.

*CONTRAST (10 stimuli).* These stimuli were constructed by varying the contrast of the noise patterns used in SPACE. Ten different contrast levels were used: 1%, 2%, 3%, 4%, 6%, 9%, 14%, 21%, 32%, and 50%. These contrast levels are relative to the contrast of the patterns used in SPACE, which is taken to be 100%.

*SEPARATION (4 stimuli).* These stimuli used the same type of noise patterns as SPACE but varied the amount of separation between contours. We generated noise patterns using cutoff frequencies of 2.8, 1.6, 0.9, 0.5, and 0.3 cycles per degree, and numbered these from 1 (smallest separation) to 5 (largest separation). The noise patterns used in SPACE correspond to separation 4; thus, we only constructed stimuli for the remaining separations 1, 2, 3, and 5. The noise patterns occupied the full stimulus extent (no aperture masking).

#### Object experiment (subject 3)

This experiment consisted of 35 stimuli, each of which corresponds to a single band-pass filtered object flashed nine times in quick succession (same temporal pattern as the stimuli in the main experiment). To construct the object stimuli, we obtained pre-segmented objects used in a previous study [63]. Objects were converted to grayscale, scaled to 200 pixels×200 pixels (9.9°×9.9°), and centered at fixation. Each image was whitened (to remove low-frequency bias) and then filtered with the custom band-pass filter (described previously). Finally, the contrast of each image was scaled to fill the full luminance range.

### Experimental design

We used a randomized event-related design to minimize anticipatory and attentional effects. Stimuli were presented in 8-s trials, one stimulus per trial. During the first 3 s of a trial, the nine images comprising a given stimulus were presented in random order at a rate of 3 images per second (duty cycle: 167-ms ON/167-ms OFF). Then for the next 5 s, no stimulus was presented.

For the main experiment, the 156 stimuli were randomly divided into four groups. In each run, the stimuli from one of the groups were presented once and in random order. To establish the baseline signal level, each run also included null trials in which no stimuli were presented (“blank” stimuli). Two null trials were inserted at the beginning and end of each run, and one null trial was inserted after every five stimulus trials. Each run lasted 6.7 minutes. Each scan session consisted of three sets of four runs (thus, each stimulus was presented three times over the course of the session). For the object experiment, the 35 stimuli were presented once and in random order in each run. Null trials were included to establish the baseline signal level as in the main experiment. Each run lasted 6.0 minutes, and each scan session consisted of ten runs.

To improve signal-to-noise ratio for the main experiment in subjects 1 and 2, two independent scan sessions were conducted. The stimulus ordering in the second session was matched to that in the first session, and the data from the two sessions were directly averaged together (after data pre-processing).

### MRI data acquisition

Functional MRI data were collected at the Stanford Center for Cognitive and Neurobiological Imaging using a 3T GE Signa MR750 scanner and a Nova 32-channel RF head coil. In each scan session, 22 slices roughly parallel to the parieto-occipital sulcus were defined: slice thickness 2.5 mm, slice gap 0 mm, field-of-view 160 mm×160 mm, phase-encode direction anterior-posterior. A T2*-weighted, single-shot, gradient-echo EPI pulse sequence was used: matrix size 64×64, TR 1.337702 s, TE 28 ms, flip angle 68°, nominal spatial resolution 2.5×2.5×2.5 mm^3^. The TR was matched to the refresh rate of the display such that there were exactly 6 TRs for each 8-s trial.

For post-hoc correction of EPI spatial distortion, measurements of the *B*_0_ magnetic field were performed. Field maps were collected in the same slices as the functional data using a 16-shot, gradient-echo spiral-trajectory pulse sequence. Two volumes were successively acquired, one with TE set to 9.091 ms and one with TE increased by 2.272 ms, and the phase difference between the volumes was used as an estimate of the magnetic field. To track slow drifts in the magnetic field (e.g. due to gradient heating), field maps were collected before and after the functional runs as well as periodically between functional runs.

### Data analysis

Voxels in each visual field map were pooled across subjects. Unless otherwise indicated, error bars represent ±1 standard error (68% confidence intervals) across voxels and were obtained using bootstrapping.

#### Data pre-processing

The first five volumes of each functional run were discarded to allow magnetization to reach steady-state. Differences in slice acquisition times were corrected using sinc interpolation. Field maps were phase-unwrapped using FSL's *prelude* utility (http://fsl.fmrib.ox.ac.uk), spatially smoothed using local linear regression [64], and then interpolated over time to estimate the field strength at the acquisition time of each functional volume. These field estimates were then used to undistort the functional volumes [65]. Motion was estimated from the undistorted volumes using utilities in SPM (http://www.fil.ion.ucl.ac.uk/spm/). Motion estimates were restricted to a manually defined 3D ellipse to avoid artifact-prone regions (e.g. near the ear canals), and were low-pass filtered at 1/90 Hz to remove high-frequency modulations that may have been caused by BOLD activations [66]. Finally, the combined effects of distortion and motion were corrected using a single cubic interpolation of the slice-time corrected functional volumes. Raw scanner units were converted to units of percent BOLD signal change by dividing by the mean signal intensity in each voxel.

For each subject, data from all scan sessions were co-registered to data from the initial scan session. This was accomplished by determining rigid-body transformations that align the functional volumes of additional scan sessions to the functional volumes of the initial scan session and incorporating these transformations into the interpolation procedure that corrects distortion and motion.

#### GLM analysis

We analyzed the time-series data from each experiment using a variant of the general linear model (GLM) commonly used in fMRI [a general review of the GLM can be found in 67]. The GLM variant that we used consisted of a flexible hemodynamic response function (HRF) characterizing the shape of the timecourse of the BOLD response, beta weights characterizing the amplitude of the BOLD response to each stimulus, polynomial regressors characterizing the baseline signal level, and global noise regressors characterizing BOLD fluctuations unrelated to the stimulus. Cross-validation (i.e. predicting left-out runs) was used to estimate the accuracy of the GLM, and bootstrapping (i.e. sampling with replacement from the runs) was used to estimate the reliability of the GLM (including error bars on response amplitudes). All subsequent analyses involved analyzing the response amplitudes estimated by the GLM. [See 11 for additional details on the GLM analysis.]

#### Second-order contrast (SOC) model

The second-order contrast (SOC) model attempts to characterize how the images shown to the subject are encoded in the response amplitudes of each voxel. In this section we describe the computations that comprise the model; in later sections we address other issues such as model fitting and model accuracy.

*Stimulus pre-processing.* The original stimulus image has a resolution of 800 pixels×800 pixels and values in the range [0,254]. The model starts by remapping the values to the range [−0.5,0.5] (which has the effect of mapping the neutral-gray background to 0) and downsampling the image to 150 pixels×150 pixels. The image is then enlarged to 180 pixels×180 pixels by padding with zeros on all sides.

*V1 energy.* The first component of the model is an adaptation of a simple V1 model that we previously developed [7]. The image is projected onto a set of isotropic Gabor filters occurring at 8 orientations, 2 quadrature phases, and a range of positions (90×90 grid). Filters occur at a single scale (appropriate since the stimuli are band-pass), with a peak spatial frequency of 3 cycles per degree and a spatial frequency bandwidth of 1 octave (full-width at half-maximum of the amplitude spectrum). Each filter is scaled such that the response to a full-contrast optimal grating is 1. Outputs of quadrature-phase filters are squared, summed, and square-rooted, analogous to the complex-cell energy model [68]. The results can be expressed as
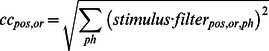
where *cc_pos_*_,*or*_ indicates the complex-cell output at a given position and orientation, *stimulus* indicates the pre-processed stimulus image, *filter_pos_*_,*or*,*ph*_ indicates the filter at a particular position, orientation, and phase, and · indicates dot product.

*Divisive normalization*. Each complex-cell output is divisively normalized by local population activity [9], [10], [69]. Local population activity is taken to be the average complex-cell output across the orientations at a given position. Formally, the operation is given by
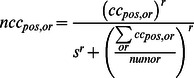
where *ncc_pos_*_,*or*_ is the normalized complex-cell output at a given position and orientation, *numor* is the total number of orientations, and *r* and *s* are parameters that control the strength of the normalization.

*Spatial summation*. The normalized complex-cell outputs are summed across orientation, yielding a measure of local contrast-energy at each position:
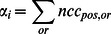
where α*_i_* is the amount of contrast-energy at position *i*. Contrast-energy is then summed across space using isotropic 2D Gaussian weights:
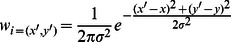
where *w_i_*_ = (*x*′,*y*′)_ is the weight at position *i* indexed by coordinates *x*′ and *y*′; *x* and *y* are parameters that control the center of the Gaussian; and σ is a parameter that controls the standard deviation of the Gaussian. Note that because of the scaling term, the sum of the weights equals one:
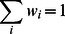



*Second-order contrast.* The summation of contrast-energy across space is not linear but involves a nonlinear squaring operation (which can be understood more generally as a rectification-type nonlinearity). Specifically, the summation is computed as
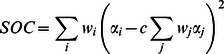
where *SOC* is the result of the summation and *c* is a parameter that controls the strength of the nonlinearity. The summation term inside the parentheses computes a spatially-weighted average of contrast-energy, and the overall expression computes spatially-weighted variance in contrast-energy. To ease interpretation, we bounded the *c* parameter between 0 and 1. When *c* is 0, the variance effect is absent and the computation is analogous to mean contrast-energy; when *c* is 1, the variance effect is strong and the computation is analogous to variance in contrast-energy; and when *c* is between 0 and 1, the computation is in between pure mean and pure variance.

*Compressive nonlinearity.* The final component of the model is a compressive power-law nonlinearity that is applied after spatial summation. The predicted response of the model is given by
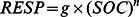
where *RESP* is the predicted response, *n* is an exponent parameter that controls the strength of the compression, and *g* is a gain parameter.

*Overall summary.* There are eight free parameters in the SOC model: *r* and *s* control the strength of divisive normalization, *x*, *y*, and σ control the region over which spatial summation occurs, *c* controls the strength of the second-order contrast effect, *n* controls the strength of the compressive nonlinearity, and *g* controls the overall gain of the predicted responses. Note that the model does not include an offset parameter. This ensures that the predicted response to a blank stimulus is 0, which is appropriate since response amplitudes reflect changes in the BOLD signal relative to a blank stimulus.

### Model fitting

We fit the SOC model to each voxel using response amplitudes to the SPACE, ORIENTATION, GRATING, PLAID, CIRCULAR, and CONTRAST stimuli. Model fitting was performed using nonlinear optimization (MATLAB Optimization Toolbox) with the objective of minimizing squared error. The predicted response to a given stimulus was obtained by computing the response of the model to each of the nine images comprising the stimulus and then taking the average across these responses.

Fitting all of the parameters in the SOC model (*r*, *s*, *x*, *y*, σ, *c*, *n*, *g*) simultaneously is computationally prohibitive. To reduce computational requirements, we determined a single set of canonical values for the *r* and *s* parameters before fitting the remaining parameters (detailed below). This strategy has the additional benefit of simplifying the interpretation of the model; for example, voxel-to-voxel differences in the overall strength of normalization can be solely attributed to differences in the *n* parameter and not differences in the *r* and *s* parameters (see Figure 9B).

Our fitting approach was as follows. To determine a single set of canonical values for the *r* and *s* parameters, we selected from each subject the ten voxels in V1 with the highest GLM cross-validation accuracy and exhaustively evaluated each combination of *r* and *s*, where *r* is chosen from {.01 .05 .1 .2 .3 .4 .5 .6 .7 1 1.5 2} and *s* is chosen from {.002 .005 .01 .02 .05 .1 .2 .5 1 2 4 8}. For each combination of *r* and *s*, we optimized *x*, *y*, σ, and *g* with *c* fixed to 0.9 and *n* fixed to 0.5, and then optimized all of these parameters simultaneously. On average across voxels, the values that produced the best fits were *r* = 1 and *s* = 0.5. We then fixed the *r* and *s* parameters to these values and fit the remaining parameters of the model for every voxel. To guard against local minima, we used a variety of initial seeds for the *c* and *n* parameters. For every combination of *c* and *n*, where *c* is chosen from {.1 .4 .7 .8 .85 .9 .95 .975 .99 .995} and *n* is chosen from {.05 .1 .2 .3 .4 .5 .6 .7 1}, we optimized *x*, *y*, σ, and *g* with *c* and *n* fixed, and then optimized all of these parameters simultaneously.

The SOC model was fit using two different resampling schemes. In the *full fit* scheme, we fit the model to the entire set of responses. This was used to derive best estimates of the parameters of the SOC model. In the *cross-validation* scheme, we fit the model using five-fold cross-validation (random selection of folds). This was used to obtain unbiased estimates of the accuracy of the SOC model.

### Model accuracy

Accuracy was quantified as the percentage of variance explained (*R* ^2^) in the measured response amplitudes by the cross-validated predictions of the response amplitudes:
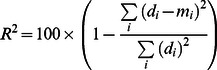
where *d_i_* indicates the *i*th measured response amplitude and *m_i_* indicates the *i*th predicted response amplitude. The *R* ^2^ value indicates the percentage of variance relative to 0 that is predicted by the model. Note that defining *R* ^2^ with respect to deviations from 0 as opposed to deviations from the mean (which is the typical statistical formulation) avoids the arbitrariness of the mean, which varies depending on the specific data points under consideration.

Model accuracy was compared to the *noise ceiling*, defined as the maximum accuracy that a model can be expected to achieve given the level of noise in the data [70], [71]. Noise ceiling estimates were obtained using Monte Carlo simulations in which a known signal and noisy measurements of the signal are generated and the expected *R* ^2^ between the signal and the measurements is calculated. In these simulations, the signal and noise are assumed to be Gaussian-distributed with parameters matched to the response amplitudes and associated error bars obtained from each voxel [see 11 for additional details]. Model accuracy was also compared to a *flat response* model that simply predicts the mean response for every stimulus.

To obtain a metric of model accuracy that is adjusted for the noise ceiling and the flat response model, we define *percent explainable variance* as
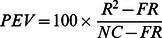
where *R* ^2^ indicates the raw performance of the model, *FR* indicates the performance achieved by the flat response model, and *NC* indicates the noise ceiling. For example, 50% explainable variance means that the amount of variance predicted by a model is halfway between the amount of variance predicted by the flat response model and the maximum amount of variance that can be predicted given the noise in the data.

As an additional assessment of model accuracy, we took the fits of the SOC model from the main experiment (full fit scheme) and predicted the response amplitudes in the object experiment. To compensate for instability in the gain of response amplitudes across scan sessions (e.g. due to imperfections in co-registration), we allowed a non-negative scale factor to be applied to the predicted response amplitudes before computing *R* ^2^ values. For fair comparison, the simulations used to estimate the noise ceiling for the object predictions also included the scale adjustment.

#### Simplified versions of the SOC model

We compared the SOC model with the CC, DN, and CSS models, which are simplified versions of the SOC model (see Figure 2C). Model fitting proceeded similarly for the simplified models, including using cross-validation to estimate model accuracy. For the CSS model, we exhaustively evaluated each combination of *r* and *s*; the remaining parameters were optimized by first optimizing *x*, *y*, σ, and *g* with *n* fixed to 0.5, and then optimizing the parameters simultaneously. We also tested an alternative version of the CSS model in which canonical values for the *r* and *s* parameters are determined before fitting the remaining parameters of the model, similar to the fitting strategy for the SOC model. The performance of this model was similar to the fully-optimized CSS model, so we report results for only the latter model. For the CC and DN models in Figure 3, since the grating stimuli do not vary in space, we fit both models assuming spatial summation at the center of the visual field. For the DN and CSS models in Figure 4, the parameters controlling the strength of divisive normalization (*r*, *s*) were fixed to the values determined in the example of Figure 3.

#### Additional control models

Besides the CC, DN, and CSS models, several additional control models were evaluated. The linear second-order (LSO) model is identical to the SOC model except that the squaring operation in the computation of second-order contrast is omitted. The flexible spatial pool (FSP) model is identical to the DN model except that the spatial scale over which normalization occurs is flexible and fit to the data. The FSP model was implemented by smoothing the map of population activity with a 2D Gaussian before divisive normalization. For each voxel we performed an exhaustive search over a range of Gaussian sizes to determine the optimal model fit. Reduced models 1–4 (RM1, RM2, RM3, RM4) are simplified versions of the SOC model (see Figure 8A). The RM1 and RM4 models use a fixed square-root nonlinearity after the computation of second-order contrast in order to maintain the scale of the computation. Versions of these models that omit the nonlinearity altogether perform even worse (results not shown).

#### Alternative methods for model selection

Cross-validation is a simple but computationally intensive technique for estimating the prediction error of a model (i.e. the error of the model on data not used to train the model). Alternatively, there are analytic methods that estimate prediction error—these include Akaike's information criterion (AIC) and Bayesian information criterion (BIC). Assuming Gaussian noise and adding a correction for small sample sizes, AIC is equal (up to additive constants that do not depend on the model) to *n* log(*SSE*/*n*)+2*k*+2*k*(*k*+1)/(*n*–*k*–1) where *n* is the number of data points, *SSE* is the sum of the squares of the residuals of the model fit, and *k* is the number of free parameters in the model. Assuming Gaussian noise, BIC is equal (up to additive constants) to *n* log(*SSE*/*n*)+*k* log(*n*). The model that minimizes AIC (or BIC) is selected as the best model [for details], [ see 64,72]. We calculated AIC and BIC for the CC, DN, CSS, and SOC models and confirmed that these metrics provide similar results to cross-validation (see Supporting Figure S1). Note that AIC and BIC are sensitive to the scale of the data, so to aid interpretation the data from each voxel were *z*-scored prior to the calculation of AIC and BIC.

#### Estimation of receptive field location

Although receptive field location can be inferred from the fits of the SOC model, it is simpler and more convenient to derive receptive field location from the fits of a purely spatial model. We used a spatial model [11] in which the predicted response is obtained by computing a weighted (isotropic 2D Gaussian) sum of an image indicating the location of the stimulus, followed by a static nonlinearity (power-law function). We fit this spatial model to each voxel using response amplitudes to the SPACE stimuli. We then derived receptive field location as a contour at two standard deviations of a 2D Gaussian that describes the response of the model to point stimuli. [This Gaussian has the same center as the model Gaussian but has a standard deviation equal to the standard deviation of the model Gaussian divided by the square root of the power-law exponent; see 11.] Receptive field locations derived in this manner are used in Figure 4 and in voxel selection procedures as described below.

#### Voxel selection

After fitting the GLM to each voxel, we selected for further consideration all voxels that have positive GLM cross-validation accuracy (indicating that responses exhibit a reliable relationship to the stimulus) and response amplitudes that are positive on average (this excludes peripheral voxels which typically exhibit negative BOLD responses to centrally presented stimuli). These voxels were then used for all subsequent analyses, with the following exceptions.

One exception was the cross-validation procedure for quantifying model accuracy in the main experiment (Figures 6–8). For this procedure we selected from each visual field map in each subject the 10 voxels with the highest GLM cross-validation accuracy (30 voxels total for each map). Note that GLM cross-validation accuracy is not biased towards any particular stimulus-response model. The number 10 was chosen to reduce computational requirements to tractable levels while also being sufficiently large to produce reliable results (e.g. see error bars on Figure 6). To verify that results do not depend on this particular threshold level, we performed an additional analysis in which we analyzed a larger number of voxels (50 voxels from each map in each subject; 150 voxels total for each map) and systematically varied the number of voxels used for model comparison (Supporting Figure S1).

Another exception was the summary of model parameters (Figure 9). For this we selected all voxels for which at least 90% of the receptive field (as derived from the simple spatial model) is contained within the stimulus bounds. This is a liberal criterion that simply excludes voxels that were inadequately sampled by the stimulus protocol.

#### Natural image simulations

To prepare a collection of natural image patches, we obtained photographs from the McGill Colour Image Database [73]. The photographs were converted to grayscale luminance values based on supplied calibration information and downsampled by a factor of two to reduce high-frequency noise. From the photographs we randomly extracted 10,000 image patches (33 pixels×33 pixels) and filtered these patches using the same band-pass filter used to construct the experimental stimuli. We then took each patch, created a version of the patch in which the phases of the Fourier components are randomized, and jointly scaled the contrast of the intact patch and the phase-scrambled patch to fill the full luminance range.

We computed the response of the SOC model to the full set of image patches. The *n* and *c* parameters of the model were matched to typical values found in V2 (*n* = 0.13, *c* = 0.993; see Figures 9b and 9c) and the *g* parameter was set such that the average response across all patches is 1. For simplicity, the parameters relating to spatial weighting (*x*, *y*, σ) were omitted from the model, and spatial constraints were enforced by simply matching the patch size to the typical receptive field size in V2 at 2° eccentricity (2.8°; see Figure 9A). We also computed the response of a control model identical to the original model except that the *c* parameter is set to 0. Setting *c* to 0 eliminates the second-order contrast effect from the SOC model.

### Public datasets and software code

Example datasets and code implementing the SOC model are provided at http://kendrickkay.net/socmodel/.

## Supporting Information

Figure S1 **Model selection using alternative metrics and different threshold levels.** As an alternative to cross-validation, we evaluated the accuracy of the CC, DN, CSS, and SOC models using Akaike's information criterion (AIC) and Bayesian information criterion (BIC). Here we plot model accuracy as a function of the number of voxels considered (voxels are selected based on GLM cross-validation accuracy; see Methods). Lines indicate the median accuracy across voxels in a given visual field map, and shaded regions indicate standard error (68% confidence intervals). Trends in model performance are consistent across metrics and are robust with respect to the number of voxels used in the model comparison.
(TIF)
