# Supplementary figures and images for: A Two-Stage Cascade Model of BOLD Responses in Human Visual Cortex
Source: PLoS Comput Biol. 2013 May 30;9(5):e1003079. doi: 10.1371/journal.pcbi.1003079 (PMC3667759; doi:10.1371/journal.pcbi.1003079)

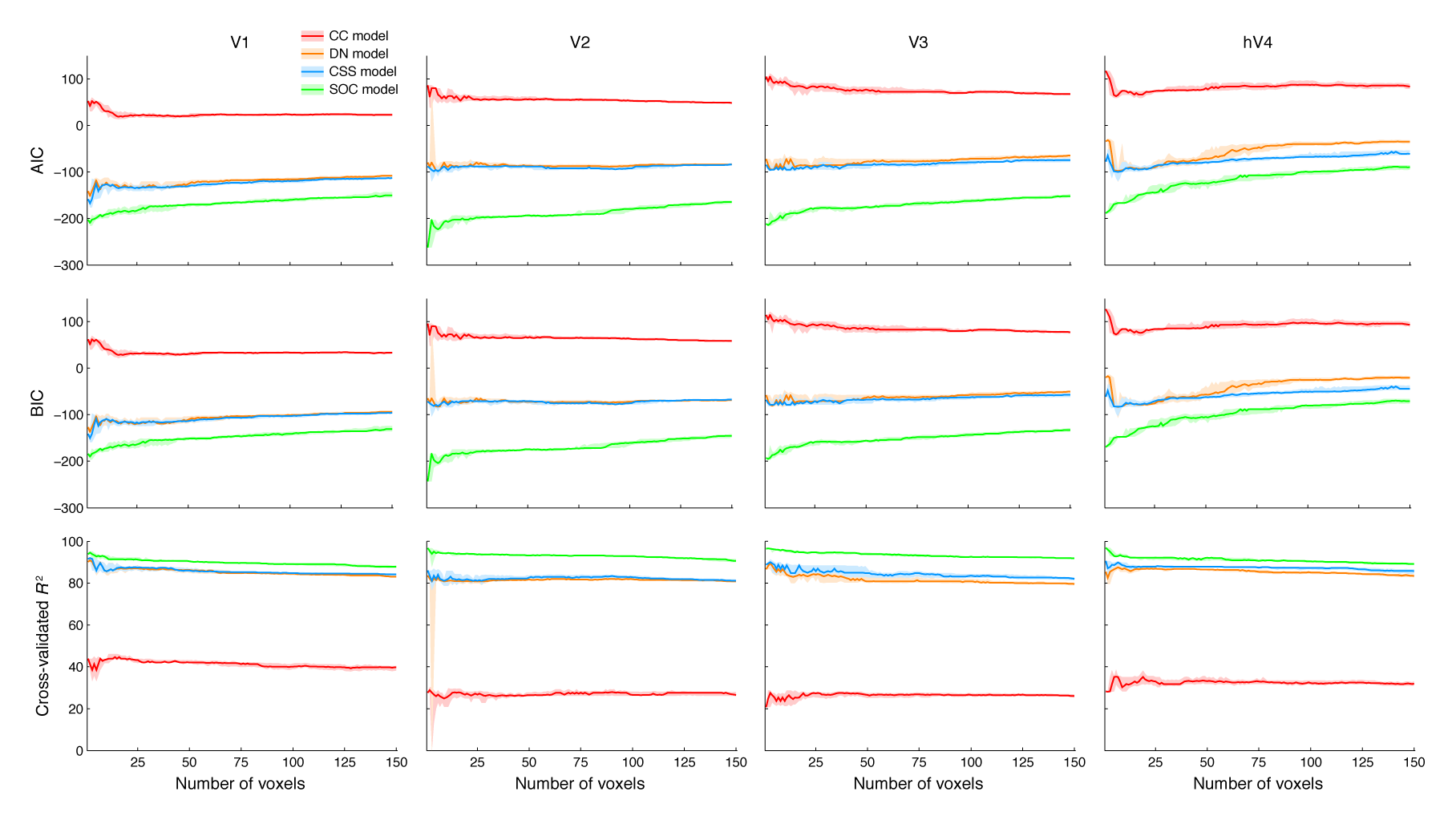

Supplement: Figure S1 — Model selection using alternative metrics and different threshold levels. As an alternative to cross-validation, we evaluated the accuracy of the CC, DN, CSS, and SOC models using Akaike's information criterion (AIC) and Bayesian information criterion (BIC). Here we plot model accuracy as a function of the number of voxels considered (voxels are selected based on GLM cross-validation accuracy; see Methods). Lines indicate the median accuracy across voxels in a given visual field map, and shaded regions indicate standard error (68% confidence intervals). Trends in model performance are consistent across metrics and are robust with respect to the number of voxels used in the model comparison. (TIF) [file pcbi.1003079.s001.tif]
